# SFB flagellin mediates cell adhesion, endocytosis and immune regulation in germ-free mice

**DOI:** 10.3389/fimmu.2025.1624092

**Published:** 2025-08-20

**Authors:** Huahai Chen, Liu Wu, Xiongyu Cao, Zongyan Li, Renjun Zhu, Xiaojing Wang, Jun Li, Zuzhang Wei, Dengfeng Yang, Yeshi Yin

**Affiliations:** ^1^ Guangxi Academy of Marine Sciences, Guangxi Academy of Sciences, Nanning, Guangxi, China; ^2^ National Key Laboratory of Non-food Biomass Energy Technology, Nanning, Guangxi, China; ^3^ Guangxi Key Laboratory of Marine Natural Products and Combinatorial Biosynthesis Chemistry, Nanning, Guangxi, China; ^4^ College of Chemistry and Bioengineering, Hunan University of Science and Engineering, Yongzhou, Hunan, China; ^5^ Key Laboratory of Comprehensive Utilization of Advantage Plants Resources in Hunan South, Yongzhou, Hunan, China; ^6^ College of Animal Science and Technology, Guangxi University, Nanning, Guangxi, China; ^7^ Guangxi Key Laboratory of Animal Reproduction, Breeding and Disease Control, Nanning, Guangxi, China; ^8^ Guangxi Veterinary Research Institute, Nanning, Guangxi, China; ^9^ Guangxi Key Laboratory of Veterinary Biotechnology, Nanning, Guangxi, China; ^10^ Key Laboratory of China(Guangxi)-Association of Southeast Asian Nations (ASEAN) Cross-border Animal Disease Prevention and Control, Nanning, Guangxi, China

**Keywords:** segmented filamentous bacteria, flagellin, germ-free mouse, bacterial adhesion, endocytosis, Th17 cell differentiation

## Abstract

**Introduction:**

Segmented filamentous bacteria (SFB) colonization dynamics are crucial for host immune regulation. Given this, the present study specifically examined the functions of SFB flagellin in bacterial adhesion, cellular internalization, and immune modulation.

**Methods:**

*Lactococcus lactis* and *Escherichia coli* were engineered to express murine and rat SFB flagellin genes. Subsequent *in vitro* co-culture experiments with intestinal epithelial cell lines (MODE-K and IEC-18) and germ-free mouse colonization assays were conducted. Bacterial counts, immunohistochemical analysis, the AAM-ISO-G1 and QAM-TH17–1 microarray systems, RNA sequencing and molecular docking were employed to assess the outcomes in this study.

**Results and discussion:**

The results of *in vitro* co-culture experiments demonstrated significantly improved bacterial adhesion capabilities mediated by SFB flagellin. Germ-free mouse colonization assays revealed prolonged fecal persistence of flagellin-expressing strains. Immunohistochemical analysis of ileal tissues showed co-localization of recombinant bacteria with the lysosomal-associated membrane protein 2 (Lamp2), confirming cellular internalization. Furthermore, *mfliC3*-expressing *Escherichia coli* exhibited active invasion into MODE-K cells. RNA sequencing analysis identified significant enrichment of Th17 cell differentiation pathways in both ileum and hepatic tissues from Lac-*rfliC3*-colonized mice. Correspondingly, the Lac-*mfliC3* group showed elevated serum levels of Th17-associated cytokines including IFN-γ, IL-23p19, IL-17A, IL-5, and IL-6 compared to controls. Molecular docking simulations revealed high-affinity interactions between SFB flagellins and endocytic regulators endophilin A2 and αM integrin. These results demonstrate that SFB flagellin mediates bacterial-epithelial interactions through dual mechanisms of adhesion potentiation and active internalization, ultimately driving Th17-mediated immune responses.

## Graphical Abstract

**(A)** Schematic illustrating SFB flagellin-mediated bacterial adhesion to epithelial cell surfaces. **(B)** Schematic depicting SFB flagellin's role in mediating cell adhesion, endocytosis, and immune regulation within germ-free mice.

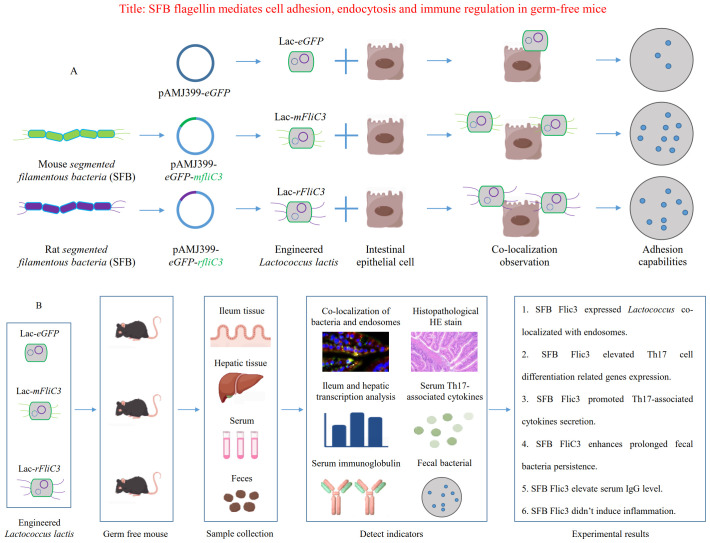

## Introduction

The gut microbiota constitutes the most extensive and complex microbial ecosystem in the human body, playing a pivotal roles in essential physiological processes including metabolic regulation and immune function. Segmented filamentous bacteria (SFB), predominantly colonizing the terminal ileum, have attracted considerable research interest due to their unique filamentous morphology and potent immunomodulatory properties. SFB colonization stimulates secretory immunoglobulin A (SIgA) production in the intestinal mucosa and drives Th17 cell differentiation (1–3), mechanisms critical for maintaining immune homeostasis (4). These commensals generally confer host beneficial, including mitigating infections by pathogens such as *Salmonella* (5) and *Staphylococcus aureus* (6), while enhancing antiviral defenses against to rotavirus and respiratory viruses (7, 8). Emerging evidence further implicates SFB in bone development and homeostasis through gut-bone axis modulation (9–11), with demonstrate roles in fracture repair (11). Notably, context-dependent SFB activities may exacerbate intestinal inflammation and strengthen autoimmune pathologies such as Crohn’s disease (12) and rheumatoid arthritis (13, 14). Collectively, SFB orchestrates acute immune-regulatory functions impacted both intestinal and systemic host health.

Although SFB are widely recognized for their essential immunomodulatory functions, their precise mechanistic contributions to host immunity and associated molecule determinants remain incompletely characterized. Ladinsky et al. demonstrated that murine SFB establish intimate interactions with intestinal epithelial cells (IECs) through surface anchoring, initiating microbial adhesion-triggered endocytosis (MATE) to deliver antigenic proteins into IECs, thereby modulating Th17 cell differentiation (15). Atarashi et al. observed that rat SFB, despite proliferating in the murine intestines, fail to colonize IEC surfaces and consequently lack Th17 regulatory capacity (16), underscoring the necessity of host-specific adhesion for SFB-mediated immune modulation.

Bacterial flagellar proteins crucially enhance microbial adhesion (17) and activate host immune responses (18), acting as key mediators of gut microbiota-host interactions that influence both microbial community dynamics and host physiology. Substantial evidence identifies these proteins as stable immune-stimulatory factors in the gastrointestinal tract, contributing to immune regulation (19, 20), β-cell functionality (21), and inflammatory bowel diseases pathogenesis (22–24). Genomic analyses of murine and rat SFB have revealed deficient metabolic gene repertoires but conserved flagellar structural genes, including four *fliC* paralogs (25–27). Our prior work validated the expression of SFB flagellin FliC3 in human and murine intestinal tissues through western blot and immunohistochemistry analyses (28, 29). Subsequent investigations demonstrated substantial genetic diversity and host specificity among SFB flagellin genes (29, 30). Notable, ELISA, ELISPOT, and RNA-seq were employed to assess the immunomodulatory function of SFB FliC3 both *in vitro* and *in vivo* (31). Furthermore, intraperitoneal administration of purified SFB FliC3 recapitulated the Th17 cell differentiation effects observed with whole SFB cells in murine models (31), suggesting potential roles for SFB flagellin in host-specific colonization and endocytic processes. This study investigates the effects of engineered SFB flagellin-expressing bacteria to cellular adhesion, endocytosis, and immune regulation in germ free mouse.

## Materials and methods

### Construction and screening of recombinant *Lactococcus lactis*

The *L. lactis* MG1363 strain and *Escherichia coli* harboring the pAMJ399-*eGFP* plasmid were maintained in our laboratory. GM17 culture medium (M17 supplemented with 0.5% glucose) and LB medium were used to culture *L. lactis* and E*. coli*, respectively. *L. lactis* was incubated at 30°C under static anaerobic conditions, while *E. coli* was cultured at 37°C with shaking at 150 rpm.

Flagellin genes derived from mouse SFB (M5I-FliC3-2, designated *mfliC3*), rat SFB (R4I-FliC3-1, designated *rfliC3*), and *Salmonella enteritidis* (*sal_fliC3*), previously cloned into the PMD18-T vector (29), were subcloned into the expression vector pAMJ399-eGFP. Competent *L. lactis* cells were prepared following established protocols with minor modifications (32). Briefly, a single *L. lactis* MG1363 colony was inoculated into 5 mL of Solution I (containing 2.5 mL 2× M17, 0.05 mL 50% glucose, 1.7 mL 50% sucrose, 0.5 mL 10% glycine, and 0.25 mL ddH_2_0), and incubated overnight at 30°C. The culture was transferred to 15 mL of Solution I at a 4% inoculation ratio, and incubated at 30°C until reaching an OD_600_ of 0.4-0.7. Cells were chilled on ice for 2 minutes, centrifuged at 4°C (5,000 rpm for 10 m), and washed twice with cold Solution II (34 mL 50% sucrose, 11.5 mL 87% glycerol, 54.5 mL ddH_2_0). After supernatant removal and tube inversion for 1 min, cells were resuspended in 1 mL of pre-chilled Solution II and incubated on ice for 30–60 min. Aliquots were dispensed into pre-cooled Eppendorf tubes and kept at -70°C for subsequent use.

Recombinant plasmid transformation into *L. lactis* MG1363 was conducted via electroporation (25 μF, 2 KV, 200 Ω). Transformants were selected on M17 agar supplemented with erythromycin (100 μg/mL). Plasmid integration was confirmed using universal vector primers P170-F (5’-CCA TTT TTG GTT GCC ATT GTT AAC GCT GCC-3’) and P170-R (5’-TAC GTA GAT CTG CTC TTC CTG CTT GAG CAT C-3’). Successful *L. lactis* transformants expressing flagellar proteins were designated Lac-*mfliC3*, Lac-*rfliC3*, and Lac-*sal_fliC3*, with the empty vector control strain named Lac-*eGFP*.

### Insertion and replacement of *E. coli fliC* with SFB *mfliC3* gene

The *E. coli* strain BW25113 was kept in our laboratory. CRISPR/Cas9-mediated genome editing replaced the native *fliC* gene in BW25113 with the SFB *mfliC3* gene. BW25113 genomic DNA was used as the template for amplifying homologous arms using primers *fliC*-up-F/*fliC*-up-R and *fliC*-down-F/*fliC*-down-R. The purified products (designated *fliC*UP and *fliC*Down), were fused via overlap extension PCR. The resultant *fliC*UD fragment was A-tailed and ligated into the pUX-T vector, generating plasmid pUX-T-*fliC*UD, which was converted into *E. coli* DH5α competent cells.

The pUX-T-flicUD plasmid served as a template to amplify the replacement/repair homologous arm using primers *mfliC*-down-F/*mfliC*-up-R. In parallel, the *tac* promoter was amplified from archived vector DNA using primers *Tac*-28a-F/*Tac*-28a-R. Using PET28a-*mfliC3* as template, the *mfliC3* sequences was amplified via primers 28a-*tac*-F/28a-*tac*-R. Purified *tac* promoter and *mfliC3* fragments were directionally ligated to generate plasmid pUX-T-28a-*tac*-*mfliC3*, which was subsequently transformed into *E. coli* DH5α competent cells.

Using pUX-T-28a-tac-*mfliC3* as template, the *Tac*-*mfliC3* fusion fragment containing promoter sequences and SFB *mfliC3* gene was amplified with primers *mfliC*-F/*mfliC*-R. Purified pUX-T-*FliC*-UD and *Tac*-*mfliC3* fragments were seamlessly assembled via Gibson cloning to generate plasmid pUX-T-*fliC*-UD-tac-*mfliC3*, which was subsequently transform into *E. coli* DH5α for downstream processing.

Competent *E. coli* BW25113 cells were prepared for electroporation. The pUX-T-Flic-UD-*tac*-*mfliC3* plasmid, alongside pCas9 and *fliC*-sgRNA, were co-electroporated into these cells. Successful SFB *mfliC3* knock-in strains were screened using primers *mfliC*-JD-F/*mfliC*-JD-R. Post-selection, temperature-sensitive plasmid curing eliminated antibiotic resistance markers, plasmid-free strain BW25113Δ*fliC*:*tac*-*mfliC3* was obtained. Primers’ sequences for genome editing are described in detail in **Supplementary Table 1**.

### Bacteria-cell co-culture for adhesion assays

MODE-K and IEC-18 cell lines maintained in our laboratory were cultured in DMEM supplemented with 15% fetal bovine serum (FBS) and 1% penicillin-streptomycin. At 80% confluence, cells were PBS-washed, detached with 0.25% trypsin-EDTA, and subcultured at a 1:3 ratio into 12-well plates. Culture medium was refreshed every 2–3 days to preserve cell viability.

Bacterial cultures were grown to mid-exponential phase (OD^600^ ≈1.0), harvested via centrifugation (5,000 ×g, 10 min), and subsequently re-suspended in sterile DMEM for individual co-culture with MODE-K and IEC-18, respectively. Cell culture medium was changed to erythromycin-supplemented DMEM prior to bacterial inoculation. After gentle agitation to ensure homogeneity, cultures were incubated in a tri-gas chamber (5% CO_2_, 2% O_2_, 37°C). Following incubation, supernatants was aspirated and the cells were washed using phosphate-buffered saline (PBS). Adherent complexes were dissociated via vigorous pipetting in 1 mL sterile H_2_O, serially diluted (10-fold increments), and plated on erythromycin-containing M17 agar for quantitative adhesion assessment.

### Recombinant *Lactococcus* administration in germ-free mice

Germ-free mouse colonization experiments were performed at Jiangsu GemPharmatech Biotechnology Co., Ltd. All procedures strictly followed institutional animal care protocols under sterile condition. Fifteen 5- to 7- week old male C57BL/6J mice were acclimated in isolators for one week and randomly allocated into five experimental groups (n=3 per group). Pre-colonization fecal pellets from individual mice underwent Gram staining to confirm axenic status.

Experimental groups were identified as follows: Group 1 (G1) received sterile PBS via oral gavage as the native control. Groups 2 to 5 (G2-G5) were administered recombinant *Lactococcus* strains Lac-*eGFP*, Lac-*sal_fliC3*, Lac-*mfliC3*, or Lac-*rfliC3*, respectively. Bacterial suspensions (2 × 10^8 CFU in 200 µL sterile PBS) were delivered on alternating (Days 1, 3, and 5) to reduce gastrointestinal stress.

Fecal samples were harvested at post-administration Days 3 and 8 for microbial load quantification. On Day 12 post-final gavage, mice under CO_2_ asphyxiation, followed by cardiac puncture for serum isolation. Intestinal luminal contents were aseptically collected for bacteriological assessment. Terminal ileum tissues (1–2 cm proximal to the cecum) and hepatic tissues were dissected and immersion-fixed in 1 mL RNAlater stabilization solution. Samples were stabilized overnight at 4°C prior to cryopreservation at -80°C.

### Detection of recombinant *Lactococcus* in fecal samples

Colonized fecal samples were harvested at designated intervals, homogenized in sterile PBS (1 mg: 30 μL), and serially diluted. Aliquots (10 µL) of the homogenate were air-dried on pre-cleaned microscope slides. Primary suspensions were further diluted (10-fold) and inoculated into erythromycin-supplemented M17 broth/agar (100 μg/mL). Cultures were incubated under strict anaerobic conditions at 30°C for 72 h, with growth kinetics monitored daily. Erythromycin-resistant colonies were quantified to determine viable bacterial loads.

### Histopathological and immunohistochemical analysis of ileal tissues

Terminal ileum segments were flushed with ice-cold PBS and fixed in 10% neutral-buffered formalin post-dissection. Tissue processing included paraffin embedding for HE (Hematoxylin and Eosin) staining and immunohistochemical evaluation. For immunohistochemistry, formalin-fixed paraffin-embedded sections were underwent sequential dehydration through xylene and graded ethanol series, following by rehydration in distilled water. Heat-mediated antigen retrieval was carried out using sodium citrate buffer (pH 6.0) or Tri-EDTA solution (pH 9.0) via microwave irradiation. Sections were blocked with 5% bovine serum albumin (BSA) for 1 h prior to overnight incubation with the primary antibodies at 4°C. After PBS washing, fluorophore-conjugated secondary antibodies were applied under light-protected condition (1 h, RT). Nuclear counterstaining with DAPI preceded final mounting with anti-fade medium. Critical protocol considerations included maintaining section hydration throughout processing and minimizing light exposure post-secondary antibody application to preserve fluorescence integrity.

### Quantification of immunoglobulins and Th17 pathway mediators

Serum immunoglobulin levels and Th17-associated cytokines were quantified using AAM-ISO-G1 and QAM-TH17–1 microarray systems, respectively, per manufacturer protocols. 1) Chip preparation: Ensure the glass slide chip is totally dry before use. 2) Antibody and sample preparation: Prepare biotin-labeled antibodies are add them to a 96-well plate, mix the diluted serum sample then with these antibodies and transfer this mixture to the antibody chip. 3) Incubation: Cover the glass slide with a sealing strip and incubate at room temperature for 1 hour. 4) Washing: Utilize the Thermo Scientific Wellwash Versa chip washer to thoroughly clean up the glass slides. 5) Scanning and detection: Employ the InnoScan 300 Microarray Scanner (Innopsys) for fluorescence signal scanning. Detect signals using the Cy3 or green channel (excitation frequency = 532 nm). 6) Data analysis: Analyze the results using either AAM-ISO-G1 or QAM-TH17–1 data analysis software.

### Comparative transcription profiling of ileum and hepatic tissues

Total RNA isolation from tissue specimens was performed using TRIzol^®^ Reagent (Invitrogen) with concomitant DNase I treatment (TaKaRa) for genomic DNA elimination. RNA integrity was checked via Bioanalyser 2100 electrophoresis (Agilent Technologies), while quantification utilized an ND-2000 spectrophotometric system (NanoDrop Technologies). RNA-seq libraries were constructed from 1 μg high-quality RNA employing the TruSeq™ RNA Sample Preparation Kit (Illumina, San Diego, CA). Polyadenylated mRNA was enriched through oligo (dT) magnetic bead selection and chemically fragmented. The cDNA synthesis, end repair, A-base addition, and ligation of Illumina-indexed adaptors followed the Illumina protocol. The libraries were size-selected for cDNA target fragments of 200–300 bps using 2% low range ultra agarose and then PCR-amplified with Phusion DNA polymerase (NEB) over 15 cycles. The libraries were quantified using a TBS380 fluorometer, and paired-end sequencing was conducted on the Illumina NovaSeq 6000 platform.

Raw paired-end sequencing reads underwent quality trimming and adapter removal using Trimmomatic with standard parameters (http://www.usadellab.org/cms/uploads/supplementary/Trimmomatic). High-quality reads were aligned to the reference genome in orientation-aware mode via TopHat (33) (http://tophat.cbcb.umd.edu/). Transcript abundance quantification employed FPKM (Fragments Per Kilobase of exon per Million mapped reads) normalization through Cuffdiff (34) (http://cufflinks.cbcb.umd.edu/). Differential gene expression analysis was conducted based on the criteria of a logarithmic fold change greater than 2 and a false discovery rate (FDR) of less than 0.05. Annotated keywords (UniProt) and enriched KEGG pathways were analyzed using the STRING database (https://cn.string-db.org/). This integrated approach elucidates transcriptome-level mechanistic disparities between ileal and hepatic tissues.

### *In vitro* endocytosis assay

Following 4 h co-culture of *E. coli* BW25113Δ*fliC*:*tac*-*mfliC3* with MODE-K cells in a 12-well plates, supernatants were aspirated and cells fixed with 4% paraformaldehyde (20 min, RT). Fixed monolayers underwent three PBS washes (10 min each) to eliminate residual fixative. Permeability with 0.2% Triton X-100 (15 min) preceded additional PBS washing. Non-specific binding was blocked with 5% BSA (30 min) prior to overnight incubation (4°C) with rabbit anti-mFliC3 polyclonal antibody in humidified chambers. After PBS washes, fluorophore-conjugated secondary antibodies were implemented light protected conditions (30 min, RT). Co-staining with anti-Lamp2 monoclonal antibody enabled endocytic compartment visualization. Final PBS rinses removed unbound antibodies, followed by DAPI nuclear counterstaining. Strict aseptic techniques and light-protected workflows were retained throughout.

### Molecular docking and pull-down assay validation

Tertiary structures of mFliC3, rFliC3, endophilin A2, and αM integrin were predicted via AlphaFold2. Predicted models underwent structural refinement using the Protein Preparation Wizard module in Schrodinger software, which included protein pre-treatment, regeneration of native ligand states, optimization of hydrogen bonds, energy minimization, and removal of water molecules. These optimized protein structures were used for protein-protein interaction simulations, employing a protein-protein docking module set to rotate the probe 70,000 times and return a maximum of 30 conformations. The lower the interaction score, indicating lower binding free energy between the ligand and receptor, the higher the binding stability. Based on these protein-protein interaction simulations, the top-ranked conformation was selected for detailed analysis.

To further validate the molecular docking results, we first constructed the eukaryotic expression plasmid pCDNA3.1-*endophilin A2*-FAG. This plasmid was transfected into 293T cells used Lipo8000 ™ (Beyotime Biotechnology, Shanghai, China). Subsequently, we specifically captured and purified the Endophili A2 protein using Magneti-Q Anti-DYKDDDK Beads (Smart-Lifesciences, Changzhou, China). Finally, pull-down binding assays were performed using our laboratory-purified mFlC3 protein (29). The mFlC3 protein was detected via immunoblotting with rabbit anti-mFlC3 polyclonal antibody (29), HRP-conjugated goat anti-rabbit IgG (Wuhan Servicebio Technology Co., Ltd., Wuhan, China), and visualized using a high-sensitivity ECL chemiluminescence kit (Wuhan Servicebio Technology Co., Ltd., Wuhan, China).

## Results

### SFB FliC3 enhances *L. lactis* adhesion

**Figure 1:**
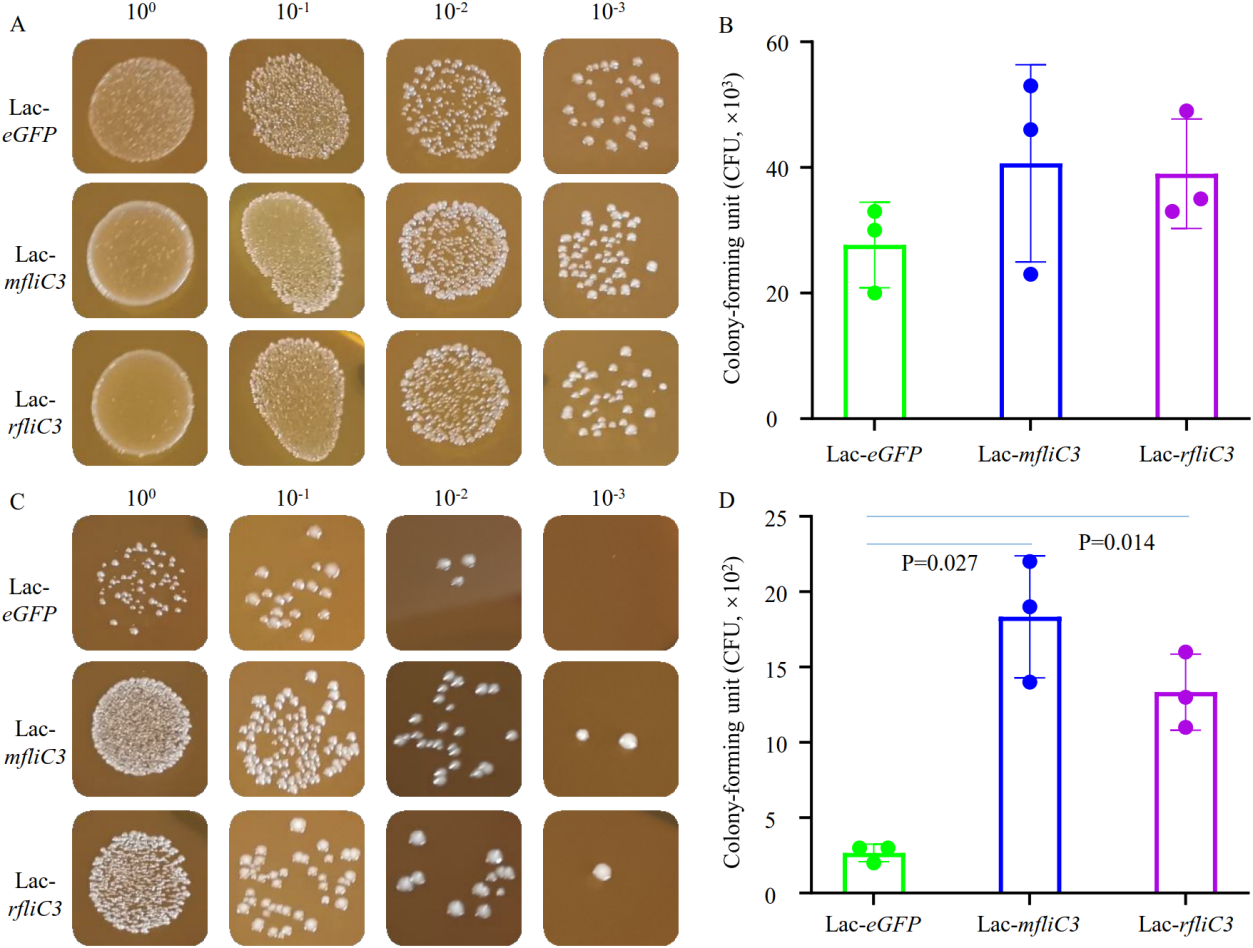
Adhesion analysis of recombinant *Lactococcus* to intestinal epithelial cells. Following 2-hour incubation with either DEME control medium or IEC-18 cells, recombinant strains (Lac-*eGFP*, Lac-*mfliC3*, Lac-*rfliC3*) were plated on M17 agar using culture supernatants or surface-adherent bacterial eluates. To quantify adherent bacteria, the co-culture supernatant was removed and the cells were gently rinsed three times with PBS. The cells were then resuspended in 1 mL of sterile water by pipetting (designating this suspension as the 100% concentration). Next, ten-fold serial dilutions of this suspension were prepared in sterile water and plated onto agar plates. Colony-forming units (CFUs) were enumerated after 18 hours of aerobic incubation at 37°C. **(A, B)** represent the bacterial colony-forming units (CFUs) after co-cultured with DEME medium. **(C, D)** represent the bacterial colony-forming unit (CFUs) after co-cultured with IEC-18. Statistical analysis was performed using Student’s t-test.

The *mfliC3* and *rfliC3* genes from SFB were subcloned into the pAMJ399-*eGFP* expression vector, generating recombinant plasmids pAMJ399-*mfliC3* and PAMJ399-*rfliC3*. Electroporation of these constructs into *L. lactis* MG1363 yield recombinant strains Lac-*eGFP* (empty vector control), Lac-*mfliC3*, and Lac-*rfliC3*. Transformants were checked through erythromycin resistance selection and Sanger sequencing of plasmid inserts. Growth curve analysis demonstrated that heterologous expression of *mFliC3* and *rFliC3* in *L. lactis* induced no significant growth impairment compared to the pAMJ399-*eGFP* control strain in M17 (**Supplementary Figure 1**) and cell culture media (**Figures 1A, B**), validating their experimental suitability for subsequent adhesion assays.

Adhesion assays involved co-culturing recombinant *Lactococcus* strains were here with murine (MODE-K) and rat (IEC-18) small intestinal epithelial cells. Following 1 h and 2 h co-culture periods, supernatants were aspirated and cells mechanically lysed in sterile PBS. Lysates were plated on erythromycin-supplemented M17 agar for quantification. Strikingly, mFliC3 and rFliC3 exhibited significantly improved bacterial adherence after 2 h exposure (**Figures 1C, D**). At 100-fold dilution, Lac-*eGFP* controls yielded only three CFUs, whereas Lac-*mfliC3* and Lac-*rfliC3* strains demonstrated robust colony formation. Under 1000-fold dilution conditions, *mfliC3*- and *rfliC3*- expressing strains retained detected colonies (1–2 CFU), while Lac-*eGFP* showed no viable counts (**Figure 1B**). Parallel results appeared in MODE-K cell-cocultures. As shown in **Supplementary Figure 2**, Lac-*mfliC3* and Lac-*rfliC3* strains exhibited markedly elevated CFU counts versus Lac-*eGFP* controls following 10-fold dilution of lysates after 1 h and 2 h co-culture periods. Moreover, ultrastructural analysis via electron microscopy confirmed recombinant *Lactococcus* adhesion to epithelial surface (**Supplementary Figure 3**). These observations demonstrate that SFB-derived mFliC3 and rFliC3 markedly potentiate adhesion to MODE-K and IEC-18 intestinal epithelial cells.

### SFB FliC3 enhances prolonged fecal bacteria persistence in mono-associated mice

To assess SFB mFliC3 and rFliC3 in enhancing intestinal colonization of recombinant *Lactococcus*, germ-free mice were subjected to oral gavage and longitudinal colonization monitoring. Fifteen germ-free male C57BL/6J mice (6–8 weeks old) were randomly allocated into five groups (n=3). And pre-colonization fecal samples underwent Gram staining verification of axenic status. Daily oral inoculations (Days 1/3/5) of 200 µL bacteria (2 × 10^8 CFU/mL) or physiological saline was used for each mouse. Fecal specimens gathered in post-gavage days 8, 13, and 17 underwent selective plating to quantify recombinant *Lactococcus* persistence. As indicated in **Table 1**; **Supplementary Figure 4**, Lac-*eGFP* controls exhibited almost undetectable fecal titers by day 8 (72 h post-final inoculation). In contrast, Lac-*mfliC3*, Lac-*rfliC3*, and Lac-*sal_fliC3* cohorts maintained significant colonization throughout the observation period. By Day 13 (8 days post-final inoculation), residual colonization persisted in the Lac-*mfliC3* group, while Lac-*rfliC3* and Lac-*sal_fliC3* cohorts maintained significant colonization. At the terminal observation timepoint (day 17, 12 days post-gavage), Lac-*mfliC3* titers fell below detection limits, whereas Lac-*rfliC3* and Lac-*sal_fliC3* groups retained persistent fecal titers. These data demonstrate that the SFB-derived mFliC3 and rFliC3 flagellins enhance recombinant *Lactococcus* intestinal persistence. Notably, rFliC3 exhibited superior colonization potential sustaining fecal excretion longer than mFliC3, suggesting enhanced ecological fitness for gastrointestinal niche adaptation.

**Table 1:** Fecal excretion of recombinant *Lactococcus* in germ-free mice.

Time	Sample name	Liquid culture								CFU on plate
		10^0^	10^-1^	10^-2^	10^-3^	10^-4^	10^-5^	10^-6^	10^-7^	
Day 8	Lac-*eGFP*	Y	Y	N	N	N	N	N	N	2.33 ± 1.89
	Lac-*mfliC3*	Y	Y	Y	Y	N	N	N	N	(3.43 ± 3.23)×10^2^
	Lac-*rfliC3*	Y	Y	Y	Y	Y	Y	Y	Y	(5.37 ± 1.41)×10^6^
	Lac-*sal_fliC3*	Y	Y	Y	Y	Y	Y	Y	Y	(3.10 ± 0.14)×10^6^
Day 13	Lac-*eGFP*	N	N	N	N	N	N	N	N	1
	Lac-mfliC3	Y	Y	N	N	N	N	N	N	6 ± 1
	Lac-*rfliC3*	Y	Y	Y	Y	Y	Y	Y	Y	(3.25 ± 0.35)×10^5^
	Lac-*sal_fliC3*	Y	Y	Y	Y	Y	Y	Y	Y	(3.35 ± 0.15)×10^5^
Day 17	Lac-*eGFP*	N	N	N	N	N	N	N	N	0
	Lac-mfliC3	Y	N	N	N	N	N	N	N	4.33 ± 0.47
	Lac-*rfliC3*	Y	Y	Y	Y	Y	Y	Y	Y	(3.13 ± 0.19)×10^6^
	Lac-*sal_fliC3*	Y	Y	Y	Y	Y	Y	Y	Y	(3.23 ± 0.05)×10^6^

Bacterial cultivation and enumeration were performed using M17 medium supplemented with 100 μg/mL erythromycin. Fecal samples were homogenized in PBS (1 mg: 30 μL) to generate the 10° stock solution. Serial ten-fold dilutions (10^-1^ to 10^-7^) were prepared in PBS from this stock. For liquid culture, 10 μL of fecal homogenate was inoculated into 190 μL of M17 medium. Turbidity development was assessed after 24 hours of anaerobic incubation at 30°C, where “Y” denotes culture turbidity and “N” indicates no visible growth. For solid-phase culture, 10 μL aliquots from serial dilutions were spread-plated on M17 agar. Colony forming units (CFU) were enumerated following 48 hours of anaerobic incubation at 30°C.

where "Y" (red) denotes culture turbidity (indicating growth), and "N" (green) indicates no visible growth.

### Lac*-rfliC3* modulates Th17 cell differentiation-associated gene expression in the murine ileum and hepatic tissues

Due to the absence of recombinant *Lactococcus* growth was observed in Lac-*eGFP* and Lac-*mfliC3* groups by day 17 post-treatment, experimental animals were humanely euthanized for biological sample collection. Intestinal and hepatic RNA samples were subsequently isolated for comparative transcription profiling. As demonstrated in **Supplementary Figure 5**, substantial differential gene expression was observed in intestinal and hepatic tissues among experimental groups. Comparative analysis showed that small intestinal samples from Lac-*mfliC3*, Lac-*rfliC3*, Lac-*sal_fliC3*, and saline control groups exhibited 67, 75, 394, and 49 significantly differentially expressed genes, respectively, relative to the Lac-*eGFP* control. Corresponding hepatic samples demonstrated 135, 115, 414, and 72 differentially expressed genes across these comparative groups.

Functional annotation of differentially expressed genes (DEGs) were performed using STRING database. UniProt keyword analysis revealed no signaling-related DEGs in sterile control. In contrast, intestinal samples from Lac-*mfliC3*, Lac-*rfliC3*, and Lac-*sal_fliC3* groups exhibiting 26, 130, and 29 signaling-related DEGs, respectively. KEGG pathway enrichment analysis identified group-specific biological signatures: while saline vs Lac-*mfliC3* comparisons showed no pathway enrichment, the Lac-*rfliC3* group displayed significant enrichment in key immunological pathways including cell adhesion, antigen processing and presentation, the intestinal mucosal immune IgA network, and Th1/Th2/Th17 cell differentiation (**Table 2**). **Supplementary Table 2** reveals distinct pathway activation patterns, with Lac-*sal_fliC3* demonstrating significant enrichment in cell adhesion and Th1/Th2 differentiation pathways, yet lacking critical enrichments were observed in Lac-*rfliC3* group including antigen processing and presentation, intestinal mucosal immune IgA network, and Th17 differentiation pathways. This differential enrichment profile highlights the unique regulatory capacity of Lac*-rfliC3* in mucosal immunity. Hepatic transcription analysis via STRING uncovered exclusive signaling features in Lac-*rfliC3* samples, with 90 signaling-related DEGs predominantly enriched in Th17-associated pathways - including Th17 differentiation and IL-17 signaling (**Supplementary Table 3**). However, these molecular signatures absent in other experimental groups (**Supplementary Table 3**).

**Table 2:** KEGG enrichment analysis of DEGs in the ileum of Lac-*rfliC3* group.

KEGG ID	Term description	No. DEGs	No. Background genes	FDR
mmu04920	Adipocytokine signaling pathway	6	71	0.039
mmu04612	Antigen processing and presentation	8	75	0.005
mmu05310	Asthma	4	24	0.025
mmu04662	B cell receptor signaling pathway	6	74	0.041
mmu04020	Calcium signaling pathway	11	191	0.023
mmu04024	cAMP signaling pathway	11	206	0.029
mmu04514	Cell adhesion molecules	12	157	0.003
mmu04060	Cytokine-cytokine receptor interaction	14	280	0.021
mmu00061	Fatty acid biosynthesis	4	18	0.020
mmu04640	Hematopoietic cell lineage	12	90	0.000
mmu05321	Inflammatory bowel disease	6	60	0.023
mmu04910	Insulin signaling pathway	8	133	0.046
mmu04672	Intestinal immune network for IgA production	7	41	0.002
mmu04978	Mineral absorption	7	51	0.004
mmu03320	PPAR signaling pathway	10	86	0.001
mmu05340	Primary immunodeficiency	8	36	0.000
mmu04974	Protein digestion and absorption	7	106	0.047
mmu04923	Regulation of lipolysis in adipocytes	6	55	0.021
mmu04924	Renin secretion	7	71	0.018
mmu04970	Salivary secretion	6	78	0.046
mmu05150	Staphylococcus aureus infection	7	108	0.050
mmu04658	Th1 and Th2 cell differentiation	7	86	0.025
mmu04659	Th17 cell differentiation	7	101	0.041
mmu05145	Toxoplasmosis	8	106	0.023
mmu04930	Type II diabetes mellitus	5	48	0.039
mmu04270	Vascular smooth muscle contraction	9	133	0.023
mmu05416	Viral myocarditis	7	74	0.020
mmu04310	Wnt signaling pathway	9	156	0.039

To assess the safety profile of these recombinant *Lactococcus* strains, ileal tissue sections were stained to evaluate immune cell infiltration, goblet cell depletion, and villus architecture integrity. As indicated in **Supplementary Figure 6E**, gastric gavage of recombinant *Lactococcus* induced no significant histopathological alterations in the upper ileum compared with the saline control. Although moderate inflammatory response and intestinal villus architecture disorder were observed in the terminal ileum of the Lac-*eGFP* group (**Supplementary Figure 6F**), the lesions induced by recombinant *Lactococcus* expressing flagellar proteins were mild (**Supplementary Figure 6F**). Overall, these recombinant *Lactococcus* expressing bacterial flagellin did not induce significant adverse effects.

### SFB FliC3-engineered *Lactococcus* enhances Th17-associated cytokine production

Serum immunoglobulin profiling and Th17-related cytokine quantification were carried out using GSM-TH17 and AAM-ISO-G1 assay systems. As shown in **Table 3**, Lac-*mfliC3*, Lac-*rfliC3*, and Lac-*sal_fliC3* groups exhibited about two-fold higher serum IgG2a and IgG3 concentrations compared with Lac-*eGFP* controls. Other immunoglobulin isotypes (IgA, IgD, IgE, and IgM) remained comparable across all experimental groups.

**Table 3:** Detection of the effects of recombinant *Lactococcus* on serum immunoglobulin and Th17 cytokines.

ProteinID	GF/egfp	salFliC/egfp	mfliC3/egfp	rfliC3/egfp
IgA	1.057	1.126	1.159	1.157
IgD	1.257	0.945	0.983	1.235
IgE	1.088	0.984	1.059	1.236
IgG1	0.903	1.436	1.786	1.302
IgG2a	1.766	1.993	2.296	2.557
IgG2b	1.016	1.337	1.424	1.183
IgG3	0.853	2.723	2.327	1.989
IgM	0.801	1.531	1.349	1.442
Kappa	0.988	1.848	1.987	1.670
Lambda	1.011	1.575	1.487	1.346
IFNg	1.040	1.278	3.170	1.317
IL-10	1.190	1.411	1.436	1.454
IL-12p70	1.145	1.470	1.463	1.287
IL-13	1.210	1.576	1.366	1.302
IL-17A	1.240	1.456	1.801	1.396
IL-17F	0.992	1.051	1.118	0.919
IL-1b	1.153	1.296	1.402	1.317
IL-2	1.136	1.279	1.285	1.370
IL-21	1.204	0.872	1.208	1.311
IL-22	0.635	0.516	0.429	0.463
IL-23p19	0.951	1.176	2.975	1.295
IL-28A	1.006	1.034	1.200	1.170
IL-4	0.954	1.115	1.079	1.008
IL-5	1.125	1.456	1.829	1.279
IL-6	1.093	1.294	1.720	2.924
MIP-3a	0.749	0.653	1.021	0.717
TGFb1	0.962	1.150	1.314	1.398
TNFa	1.103	1.313	1.500	1.311

The three gradient colors of red, yellow, and green represent the magnitude of the fold change. Red represents decrease, green represents increase.

Cytokine profiling revealed strain-specific activation patterns: The Lac-*mfliC3* group demonstrated striking elevations (>1.5-fold vs Lac-*eGFP* control) in IFNγ, IL-17A, IL-23p19, IL-5, IL-6, and TNFα levels. In contrast, Lac-*rfliC3* administration selectively enhanced IL-6 production, while comparative analysis against Lac-*sal_fliC3* group identified exclusive upregulation of IL-13 in the Lac-salFliC3-treated mice. These differential cytokine signatures delineate strain-specific immunomodulatory capacities within the Th17 axis.

### Co-localization analysis of SFB Flic3-engineered *Lactococcus* with endocytosis biomarker Lamp2

**Figure 2:**
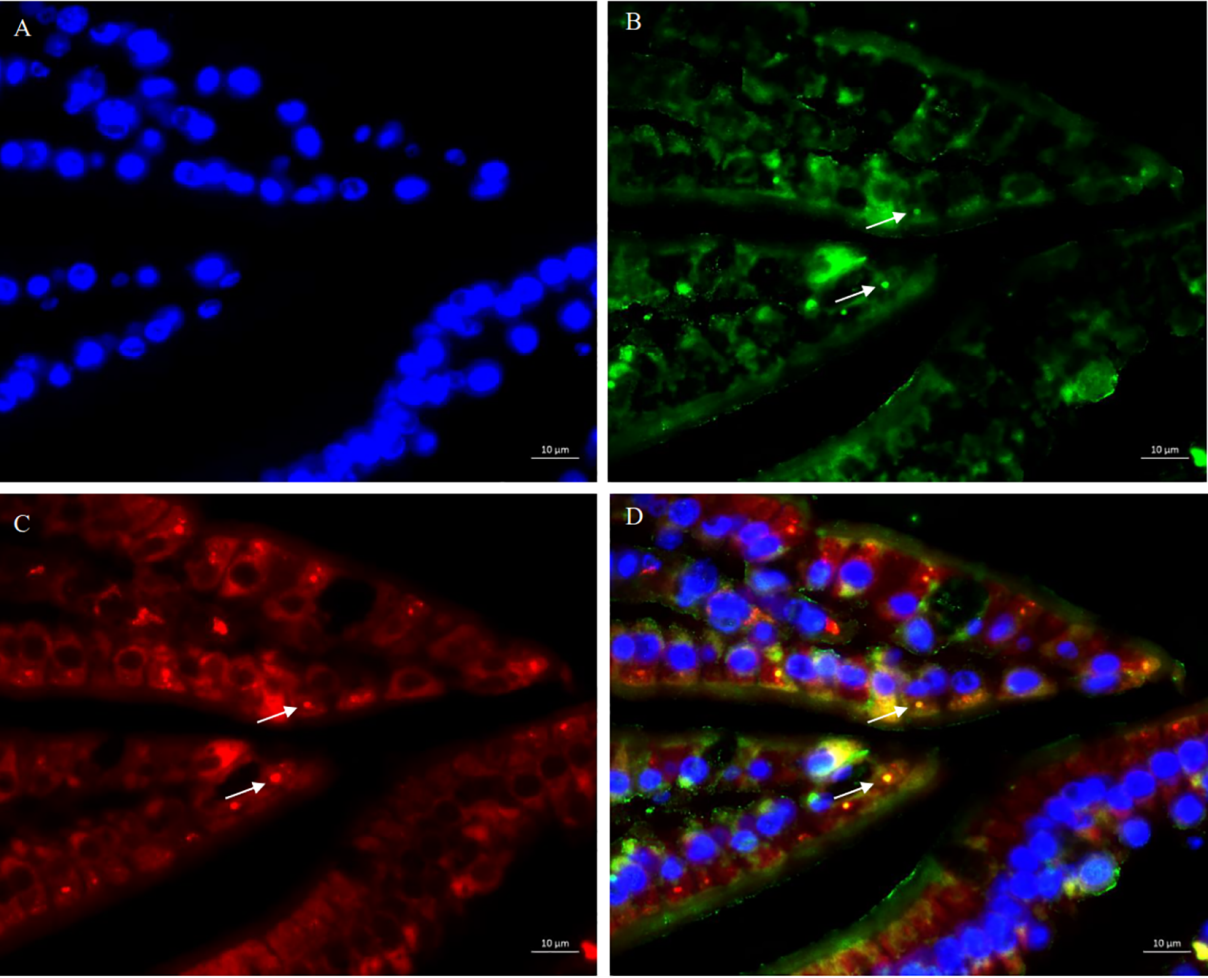
Co-localization analysis of SFB rFliC3 with endosomal marker LAMP2. Ileal tissues were fixed in 4% paraformaldehyde and sequentially immune-stained with rabbit anti-SFB FliC3 primary antibody, anti-LAMP2 antibody, and FITC-conjugated goat anti-rabbit IgG secondary antibody. Nuclei were counterstained with DAPI. Fluorescence imaging was performed using an upright microscope (Zeiss Axio Imager Z2). **(A)** DAPI staining results of ileal tissue; **(B)** Anti-SFB FliC3 antibody staining results of ileal tissue; **(C)** Anti-LAMP2 antibody staining results of ileal tissue; **(D)** Merged images of **(A–C)** demonstrating co-localization signals (yellow) of FliC3 and LAMP2.

Lysosome-associated membrane protein (LAMP1/2) is crucial for the lysosome function and stability (35). Given that SFB antigens were internalizes into cells through endocytosis to exert immune regulatory effects, and the lysosome marker LAMP2 associated with cell adhesion is involved in this process (15), we investigated the endocytic capacity of SFB *fliC3*-expressing *Lactococcus* by using dual immunofluorescence staining with anti-SFB FliC3 polyclonal antibody and anti- Lamp2 monoclonal antibody (supplied by DSHB, ABL-93). Immunohistochemistry analysis revealed significant co-localization between *rfliC3*-expressing *Lactococcus* strains and Lamp2^+^ endosomes (**Figure 2**). This spatial association suggests functional conservation with native SFB in host cell internalization mechanisms.

### MODE-K cell internalization of mFliC3 engineered *E. coli*

Since Lac-*mfliC3* was not detected in terminal mouse fecal samples (**Supplementary Figure 4**) and the co-localization fluorescence signals were weak following co-culture with MODE-K cells (**Supplementary Figure 7**), we therefore assessed its cellular internalization potential using co-culture experiments with engineered *E. coli*. The BW25113 strain was genetically engineered via *fliC* locus replacement with SFB-derived *mfliC3*. Despite similar growth dynamics across these strains (**Supplementary Figure 8**), phenotypic characterization revealed subtle morphological alterations: deletion of *fliC* slightly reduced colony size while maintaining defined margins (**Supplementary Figure 9**). Successful knock-in of *mfliC3* preserved colony size but induced a distinct undulating morphology, contrasting with the architecture of the parental strain.

**Figure 3:**
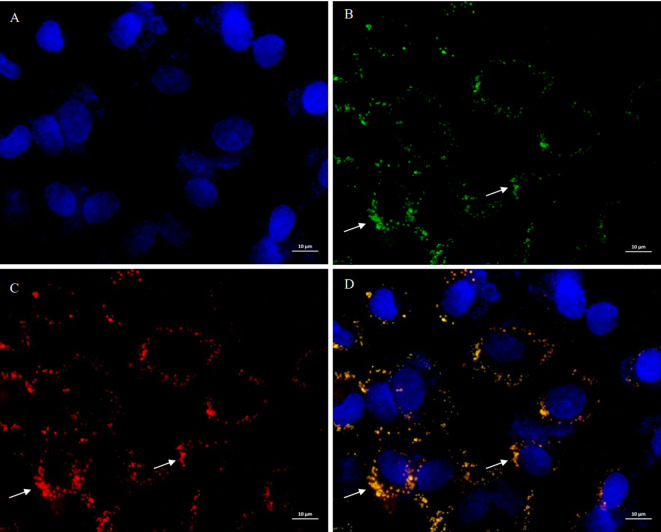
Subcellular localization of mFliC3 in MODE-K murine intestinal epithelial cells. MODE-K cells were incubated with *mfliC3*-expressing *E. coli* (BW25113*ΔfliC*:*tac-mfliC3*) for 12 hours. Following PBS washing, triple immunofluorescence staining was performed using DAPI nuclear counterstain (blue), anti-mFliC3 antibody (green), and anti-LAMP2 endosomal marker (red). **(A)** DAPI staining results of cells; **(B)** Anti-SFB FliC3 antibody staining results of cells; **(C)** Anti-LAMP2 antibody staining results of cells; **(D)** Merged images of **(A–C)** demonstrating co-localization signals (yellow) of FliC3 and LAMP2. **(D)** shows merged fluorescence channels. Specimens were mounted in anti-fade medium and imaged by confocal microscopy (scale bar: 10 μm).

Further PCR validation and Sanger sequencing confirmed successful site-specific allelic replacement (**Supplementary Figure 10**). Following 12-h co-culture of MODE-K cells with BW25113Δ*fliC*: *tac mfliC3*, dual immunofluorescence microscopy using (1) SFB FliC3-specific and (2) Lamp2-targeting antibodies revealed bacterial co-localization with Lamp2^+^ endosomal compartments (**Figure 3**). Notably, intracellular mFliC3 epitopes were detected, suggestive of flagellin translocation through endocytic machinery in intestinal epithelial cells.

### Structural prediction and molecular docking of SFB FliC3 interactions with Endophilin-A2 and αM integrin

**Figure 4:**
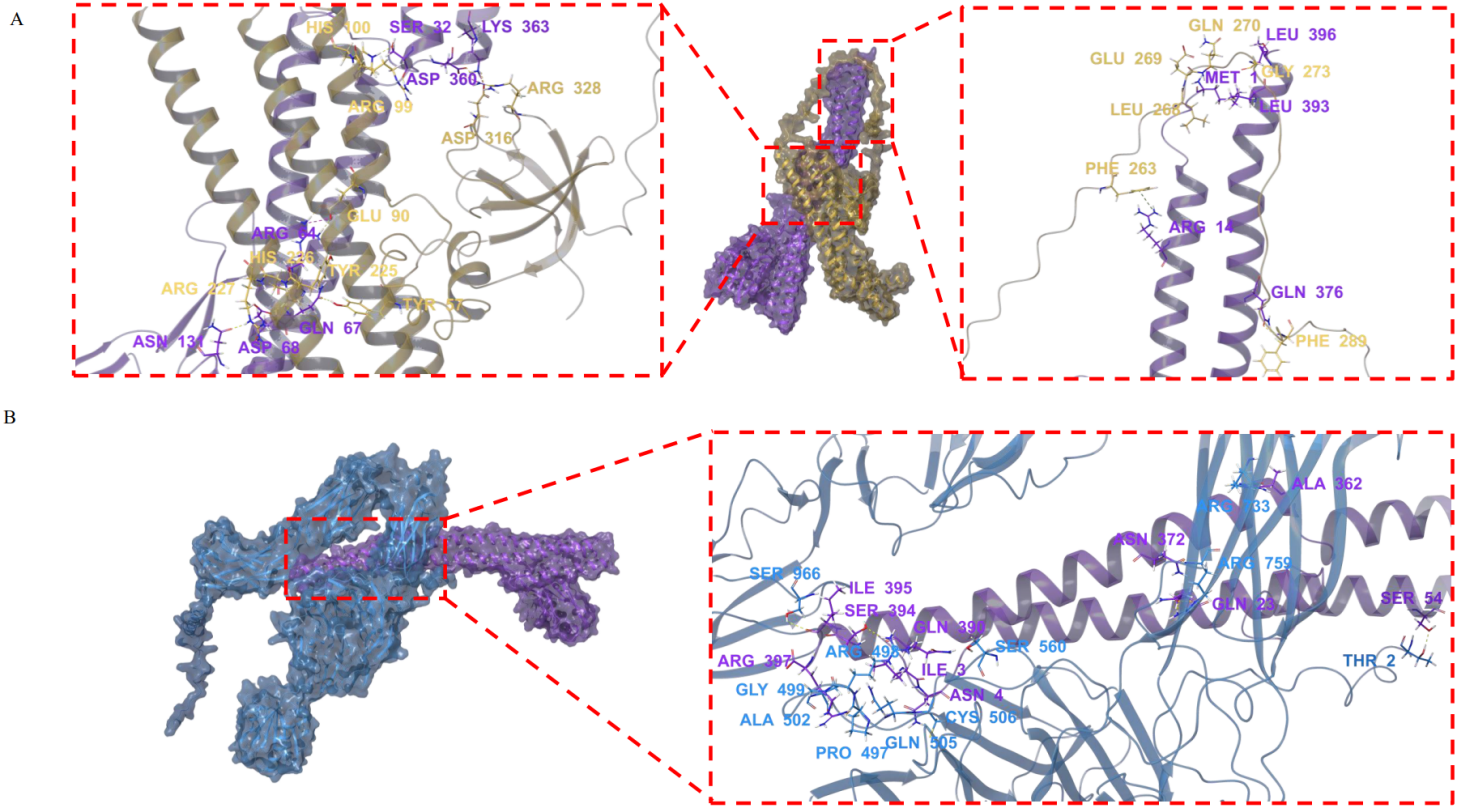
Molecular docking analysis of mFliC3 interactions with Endophilin A2 and αM integrin. The nucleotide sequence of M5I-FliC3-2 (KX658895.1), the protein sequences of Endophilin A2 (NP_038692.1) and αM integrin (P05555.2) were download from NCBI database. The tertiary structures of these genes were predicted by using AlphaFold2. Molecular docking analysis was performed then using the Protein Preparation Wizard module in Schrodinger software. Structural models were energy-minimized and visualized using PyMOL. **(A)** Predicted binding interface between mFliC3 (purple) and Endophilin A2 (yellow). **(B)** Molecular interaction profile of mFliC3 (purple) with αM integrin (blue).

By integrating AlphaFold2-derived structural predictions with Schrödinger-based molecular docking simulations, we established SFB FliC3’s ability to form stable intermolecular complexes with Endophilin-A2 and αM integrin. The predominant docking conformations demonstrated substantial binding stability for mFliC3-Endophilin-A2 (PIPER score: -351.268; interaction energy: -1023.01 kcal/mol) and mFliC3-αM integrin (PIPER score: -253.862; interaction energy: -1036.51 kcal/mol). Interface characterization revealed three distinct interaction modes between mFliC3 and Endophilin-A2: Arg328 formed a hydrogen bond with Asp360, Asp316 involved in dual interactions (hydrogen bond and salt bridge) with Lys363, and Phe289 established a hydrogen bond with Gln376 (**Figure 4A**). Similarly, αM integrin interactions featured Ser966 hydrogen-bonded with Ile395, while Arg759 formed three molecular contacts - one with Asn372 and two with Gln23 (**Figure 4B**). These structural analyses illuminate the multivalent binding architecture underlying mFliC3-mediated host protein engagement in cellular systems. The pull-down assay further validated the interaction between Endophilin-A2 and mFliC3 (**Supplementary Figure 11**).

## Discussion

### Evolutionary and functional characterization of SFB flagellin-mediated host-microbe interactions

Bacterial adhesion is a critical determinant for host colonization, tissue invasion, and biofilm formation. While microbial colonization mechanisms remain incompletely understood, emerging evidence implicates flagellar components as key mediators. Comparative genomic analyses reveal conserved roles of flagellin genes in enteric colonization across diverse pathogens including *Salmonella Enteritidis* (36), *Campylobacter jejuni* (37), and *E. coli* (38, 39) – a paradigm recently extended to gut commensals (40). Our phylogenetic studies of rodent-specific SFB flagellins demonstrated host-driven evolutionary divergence, with murine and rat variants forming distinct clades at both nucleotide and protein levels (29). Functional characterization revealed mFliC3/rFliC3 equivalently enhanced microbial adhesion to murine (MODE-K) and rat (IEC-18) intestinal epithelia *in vitro* (**Figure 1**), contrasting with predicted host specificity based on evolutionary patterns. This phenotypic divergence from phylogenetic predictions suggests micro-environmental factors (e.t., host mucosal proteases) may modulate flagellin functionality *in vivo*, as demonstrated by rFliC3’s accelerated degradation versus mFliC3 in mucosal protein co-incubation assays (29).

### Dual-phase dynamics of flagellin-engineered *Lactococcus* colonization and immune regulation

Both *mfliC3* and *rfliC3*-expressing *Lactococcus* strains demonstrated enhanced intestinal persistence *in vivo*, with *rfliC3* variants exhibiting prolonged fecal shedding (detectable until day 12 post-gavage cessation) compared to *mfliC3* (day 8) and empty vector controls (day 3). This colonization pattern inversely correlated with host immune activation: Multiplex serum analysis revealed robust Th17-polarized responses in mFliC3-administered mice, including elevated IL-17A, IL-23p19, and TNFα versus controls (**Table 3**). In contrast, rFliC3 exposure elicited selective IL-6 upregulation without broad cytokine induction. We propose this differential immunogenicity drives strain-specific clearance kinetics (38, 41, 42). The relative low level of IL-6 concentration in the mFliC3 group maybe due to the reduced colonization of Lac-*mflic3* that was verified by undetectable fecal colonies and absent mFliC3-LAMP2 co-localization by day 17. This aligns with established flagellar immunobiology: While flagellins facilitate mucus penetration through adhesive domains, their recognition as MAMPs triggers TLR5/NF-KB-mediated elimination - a self-limiting mechanism explaining SFB’s age-dependent colonization dynamics (43). Given the persistent colonization observed for Lac-*sal_fliC3* in this study (**Table 1**; **Supplementary Figure 4**), the relatively low IL-6 concentration in the Lac-*sal_fliC3* group may reflect the dynamic fluctuations of plasma IL6 levels following recombinant *Salmonella* flagellin administration. As reported by Rolli et al., plasm IL-6 peaks within one hour post-stimulation by *Salmonella* flagellin and declines steady, returning to baseline within three hours (44). However, further investigations is required to determine whether rFliC3 can sustain IL-6 production in serum.

### Temporal resolution of Th17-associated transcriptional and translational responses

This apparent incongruity between serum cytokine elevations and transcription profiles may reflect sampling chronology. By day 13 post-inoculation, recombinant bacterial loads had substantially reduced, reaching undetectable levels in fecal samples by the terminal sampling timepoint (day 17). Consequently, ileal and hepatic transcriptional signatures analyzed at day 17 likely represent post-clearance homeostasis, with Th17 pathway activation having returned to baseline levels.

### Multilateral immune modulation by rFliC3-engineered *Lactococcus*

Our investigation demonstrates that Lac-rFliC3 administration in gnotobiotic mice induces Th17 pathway enrichment in ileum and hepatic transcriptions, corroborating previous findings on SFB flagellin-mediated Th17 differentiation. Immunofluorescence co-localization of Lac-rFliC3 with LAMP2+ ednosomes substantiates its cellular internalization capacity. Paradoxically, serum proteomics analysis revealed selective IL-6 evaluation without broad Th17-associated cytokine induction, despite pronounced transcriptions alterations. This dichotomy may stem from multifactorial regulation: (1) enhanced rFliC3 proteolytic susceptibility in mucosal environments weakens its immune regulatory function (29); (2) competing immune activation evidenced by ileal enrichment of Th1/Th2 differentiation and primary immunodeficiency pathways; (3) network-level counter-regulation through cytokine cross-talk mechanisms.

### Mechanistic insights into SFB flagellin-mediated endocytic trafficking

Endocytosis serves essential roles in cellular homeostasis by mediating extracellular substance uptake and intercellular communication. While clathrin-mediated endocytosis is the canonical pathway, emerging research has characterized alternative internalization mechanisms, including fast endophillin-mediated endocytosis (FEME) (45). This clathrin-independent pathway, orchestrated by endophilin A2, enables rapid ligand-receptor complex internalization following specific surface receptor activation (46). The FEME machinery exhibits three hallmark features (45): (1) membrane remodeling, endophilin A2 induces localized plasma membrane curvature through its BAR domain-mediated lipid binding; (2) cargo selectivity, phosphorylation-dependent recognition of activated receptors ensures substrate specificity; (3) vesicle maturation, coordinated interactions with dynamic GTPase and CDC42 small GTPase facilitate vesicle scission and cytoskeletal transport.

Pioneering electron tomography studies by Ladinsky et al. revealed that SFB orchestrates antigen transfer through plasma membrane-associated endocytic vesicle formation at the host-microbe interface, rather than direct cellular invasion (15). This clathrin-independent process requires dynamic GTPase and CDC42-mediated actin remodeling, suggesting evolutionary convergence with fast endophilin-mediated endocytosis (FEME) mechanisms. Our molecular docking data demonstrate high-affinity interactions between SFB FliC3 and two key FEME components further supports FEME-like internalization (**Figure 3**). While *Salmonella* flagellin utilizes Hsp90-Hsp70-αM/β2 integrin complexes for endocytosis, SFB appears to employ distinct machinery, potentially involving: Cytoskeletal regulators (co-precipitated with FliC3 in prior studies), heat shock protein networks, integrin-mediated adhesion platforms. However, based on current experimental evidence, it remains undetermined whether endocytosis of SFB flagellin depends on clathrin-associated pathway. The precise endocytosis mechanism requires further experimental elucidation.

In conclusion: as illustrated in **Graphical abstract**, SFB flagellin FliC3 enhances bacterial epithelial adhesion and activates Th17-associated immune signaling. Mechanistically, FliC3 may undergo endophilin A2-mediated endocytosis to exert immunomodulatory effects, as supported by molecular docking and Lamp2 co-localization evidence. Further studies employing targeted genetic manipulation of SFB flagellar components in axenic cultures, combined with longitudinal sampling in conventional and gnotobioitc murine models following recombinant strain administration, will elucidate the spatiotemporal dynamics of FliC3-mediated immunoregulation.

## Data Availability

Sequence data that support the findings of this study have been deposited in the NCBI SRA database with the primary accession code PRJNA1109453 and PRJNA1108060.
