# Supplementary material for: SFB flagellin mediates cell adhesion, endocytosis and immune regulation in germ-free mice
Source: Front Immunol. 2025 Aug 20;16:1624092. doi: 10.3389/fimmu.2025.1624092 (PMC12405183; doi:10.3389/fimmu.2025.1624092)
Supplement: Supplementary file 1 [file DataSheet1.docx]

**Supplementary for**

**SFB flagellin mediates cell adhesion, endocytosis and**

**immune regulation in germ-free mice**

Huahai Chen^1, 2, 3, 4, 5^, Liu Wu^6, 7^, Xiongyu Cao^6, 7^, Zongyan Li^6, 7^, Renjun Zhu^6, 7^,

Xiaojing Wang^6, 7^, Jun Li^8, 9, 10^, zuzhang Wei^6, 7^, Dengfeng Yang^1, 2, 3*^, Yeshi Yin^6, 7*^

^1^Guangxi Academy of Marine Sciences, Guangxi Academy of Sciences, Nanning, Guangxi, China, ^2^National Key Laboratory of Non-food Biomass Energy Technology, Nanning, Guangxi, China, ^3^Guangxi Key Laboratory of Marine Natural Products and Combinatorial Biosynthesis Chemistry, Nanning, Guangxi, China, ^4^College of Chemistry and Bioengineering, Hunan University of Science and Engineering, Yongzhou, Hunan, China, ^5^Key Laboratory of Comprehensive Utilization of Advantage

Plants Resources in Hunan South, Yongzhou, Hunan, China, ^6^College of Animal Science and Technology, Guangxi University, Nanning, Guangxi, China, ^7^Guangxi Key Laboratory of Animal Reproduction, Breeding and Disease Control, Nanning, Guangxi, China, ^8^Guangxi Veterinary Research Institute, Nanning, Guangxi, China, ^9^Guangxi Key Laboratory of Veterinary Biotechnology, Nanning, Guangxi, China, ^10^Key Laboratory of China(Guangxi)-ASEAN Cross-border Animal Disease Prevention

and Control, Nanning, Guangxi, China.

**Corresponding Authors**

Yeshi Yin, Email [yinyeshi@gxu.edu.cn](mailto:yinyeshi@gxu.edu.cn) or [yinyeshi@126.com](mailto:yinyeshi@126.com);

Dengfeng Yang, Email [dengfengyang@163.com](mailto:dengfengyang@163.com).

**Supplementary Figure 1. Growth kinetics of recombinant *Lactococcus* strains in M17 broth.** Recombinant *Lactococcus* strains (Lac-*eGFP*, Lac-*mFliC3*, and Lac-*rFliC3*) were cultured in M17 medium supplemented with erythromycin (100 μg/mL), and growth kinetics were monitored by measuring optical density at 600 nm (OD₆₀₀) at the indicated time points.


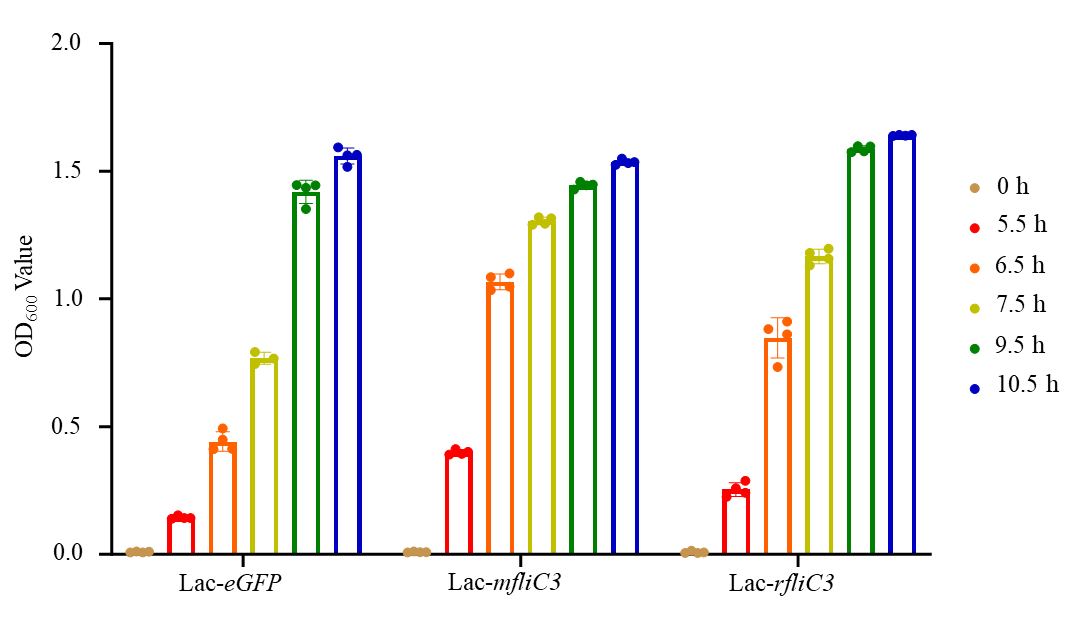


**Supplementary Figure 2. Adhesion of recombinant *Lactococcus* to MODE-K cells.** Recombinant *Lactococcus* strains (Lac-*eGFP*, Lac-*mFliC3*, and Lac-*rFliC3*) were cultured to mid-exponential phase (OD₆₀₀ ≈1.0), re-suspended in sterile DMEM, and individually co-cultured with MODE-K cells. After incubation, supernatants were aspirated and the cells were washed with phosphate-buffered saline (PBS). Adherent complexes were dissociated by vigorous pipetting in 1 mL sterile H2O, serially diluted (10-fold increments), and plated on M17 agar supplemented with erythromycin for bacterial adhesion quantification.


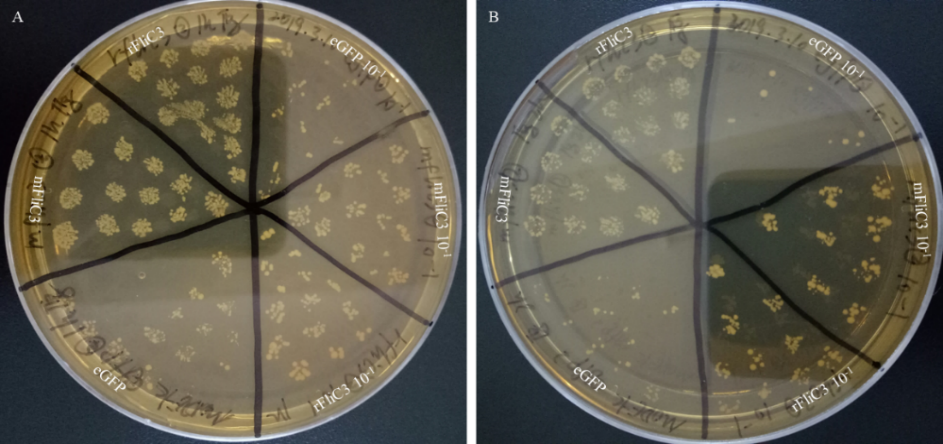


**Supplementary Figure 3. Observation of the adhesion between *Lactococcus* and IEC cells using electron microscopy.** Following co-culture of recombinant *Lactococcus* strains (Lac-*eGFP*, Lac-*mfliC3*, and Lac-*rfliC3*) with intestinal epithelial cells (IECs), supernatants were aspirated and cells were washed with ice-cold PBS. The IECs were then fixed and processed for electron microscopy. A. B and C represent the Lac-*eGFP* group, Lac-*mfliC3* group, and Lac-*rfliC3* group, respectively.

**
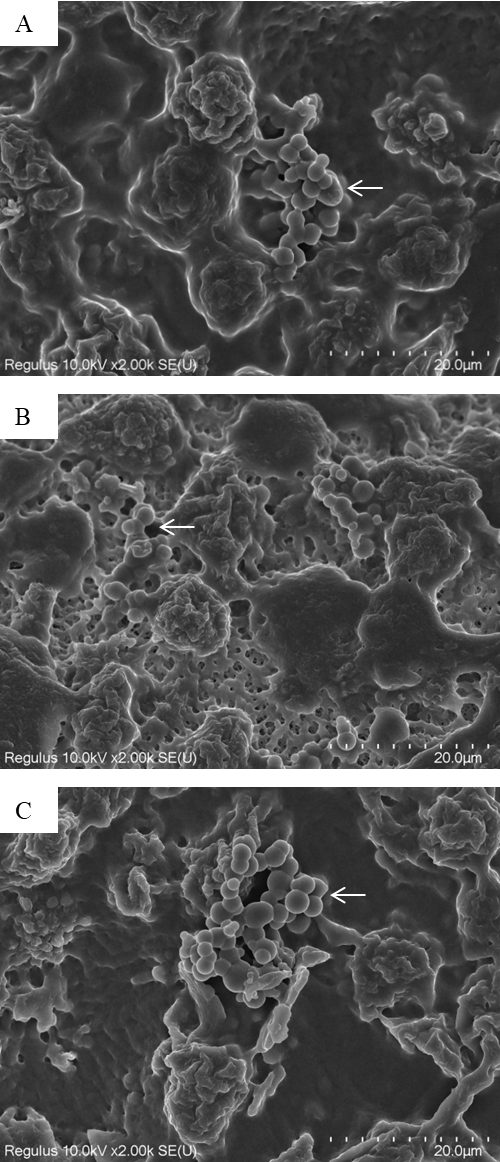
**

**Supplementary Figure 4. Detection of recombinant *Lactococcus* excretion in fecal samples.** Fecal samples were collected from mice on days 8, 13, and 17 post-administration. Specimens were serially diluted in PBS and plated on M17 agar plates. Following overnight incubation at 37°C under aerobic conditions, bacterial colonies were enumerated. A, B, and C display results from days 8, 13, and 17, respectively.

**
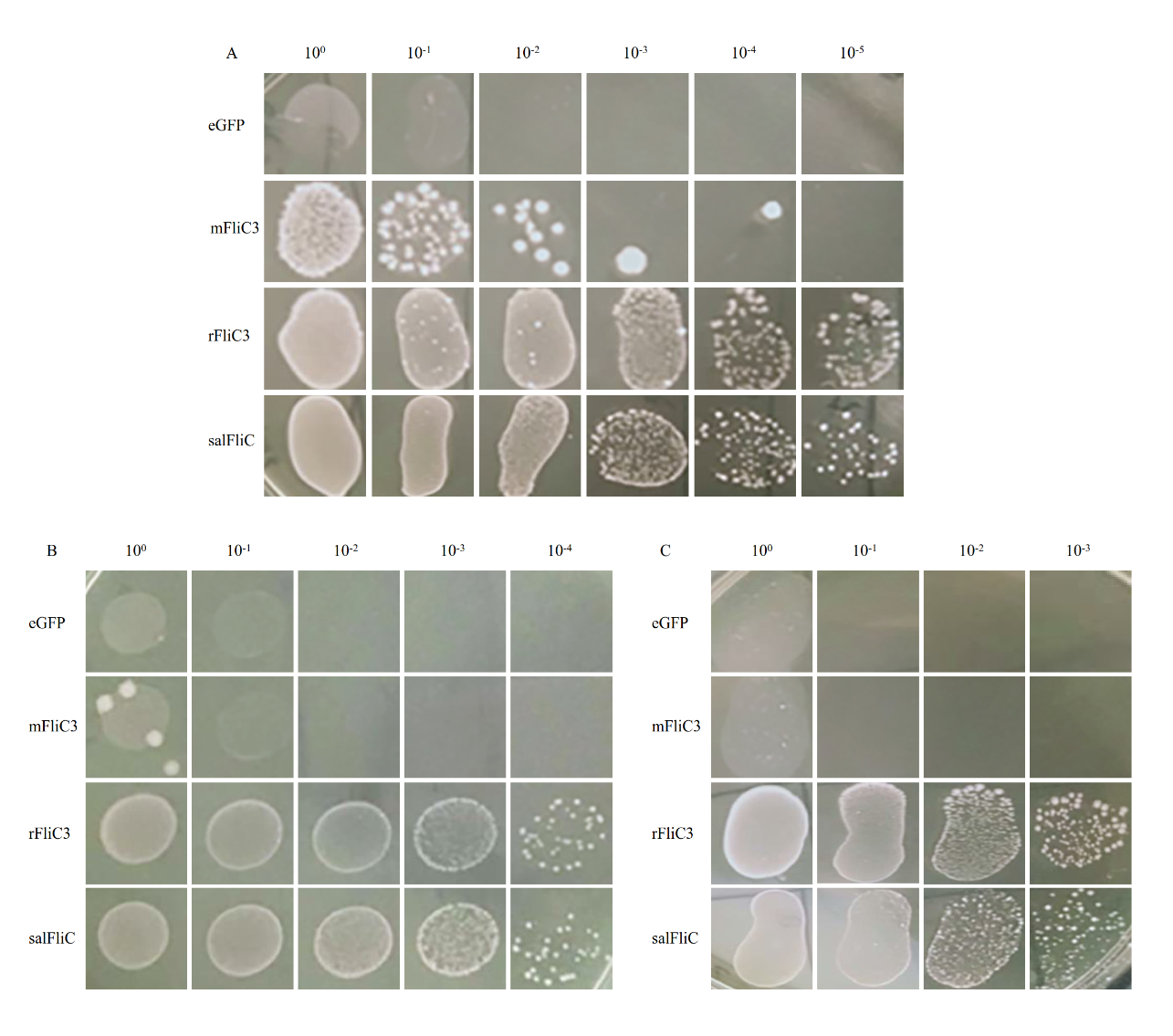
**

**Supplementary Figure 5. Statistics analysis of the number of DEGs.** At the experimental endpoint of the germ-free mouse study, ileal and hepatic tissues were collected for RNA extraction and transcriptome analysis. The different expressed genes (DEGs) were identified by compared to the Lac-*eGFP* group. SI represent ileum samples, and Liver represent liver samples.


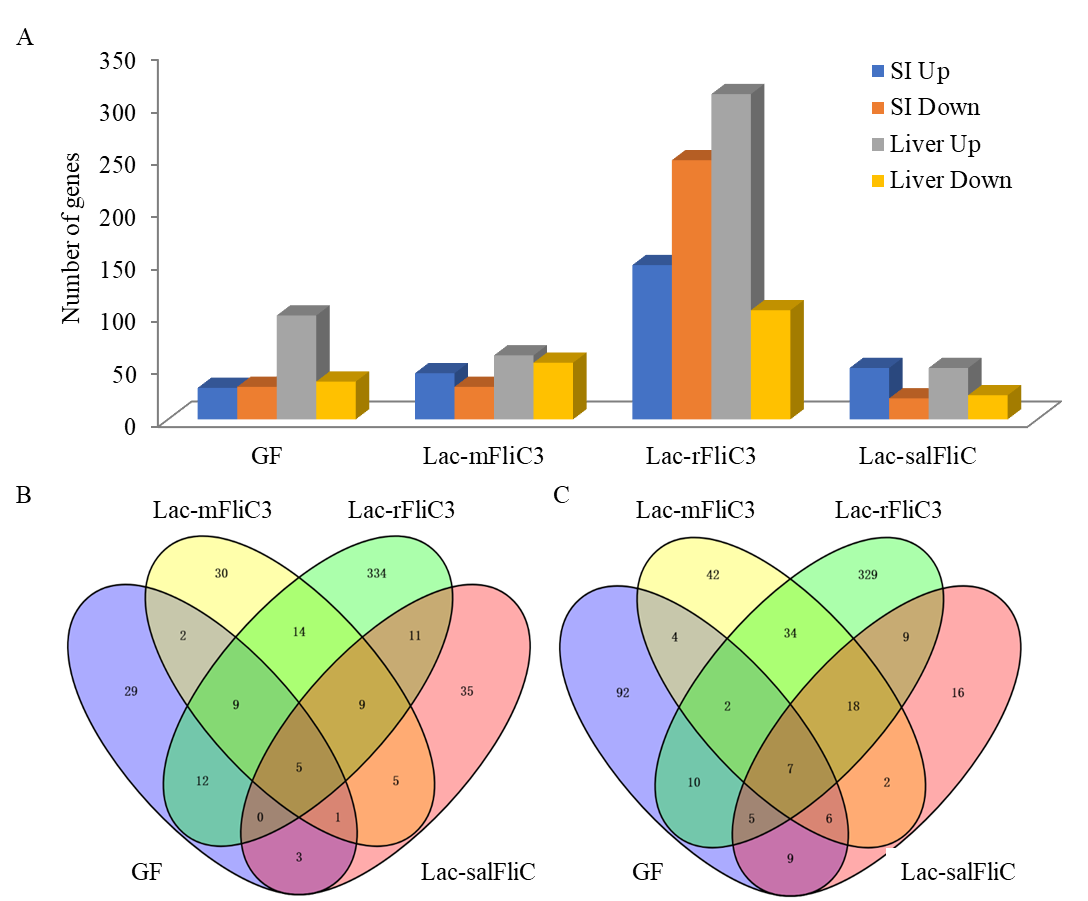


**Supplementary Figure 6. Histopathological analysis of terminal ileum tissues by H&E staining.** Terminal ileal tissues were harvested at the experimental endpoint of the germ-free mouse study, fixed, and processed for histopathological assessment. Panels A-D correspond to representative sections from the Lac-*eGFP*, Lac-*sal_fliC*, Lac-*mfliC3*, and Lac-*rfliC3* groups, respectively. E and F present blinded histopathological scores for immune cell infiltration, goblet cell depletion, and villus architecture in the upper and lower segments of the ileum, respectively. (PS: physiological saline group). A score of 0 to 3 represents severity, 0 represents lightest, and 3 represents most severe.

**
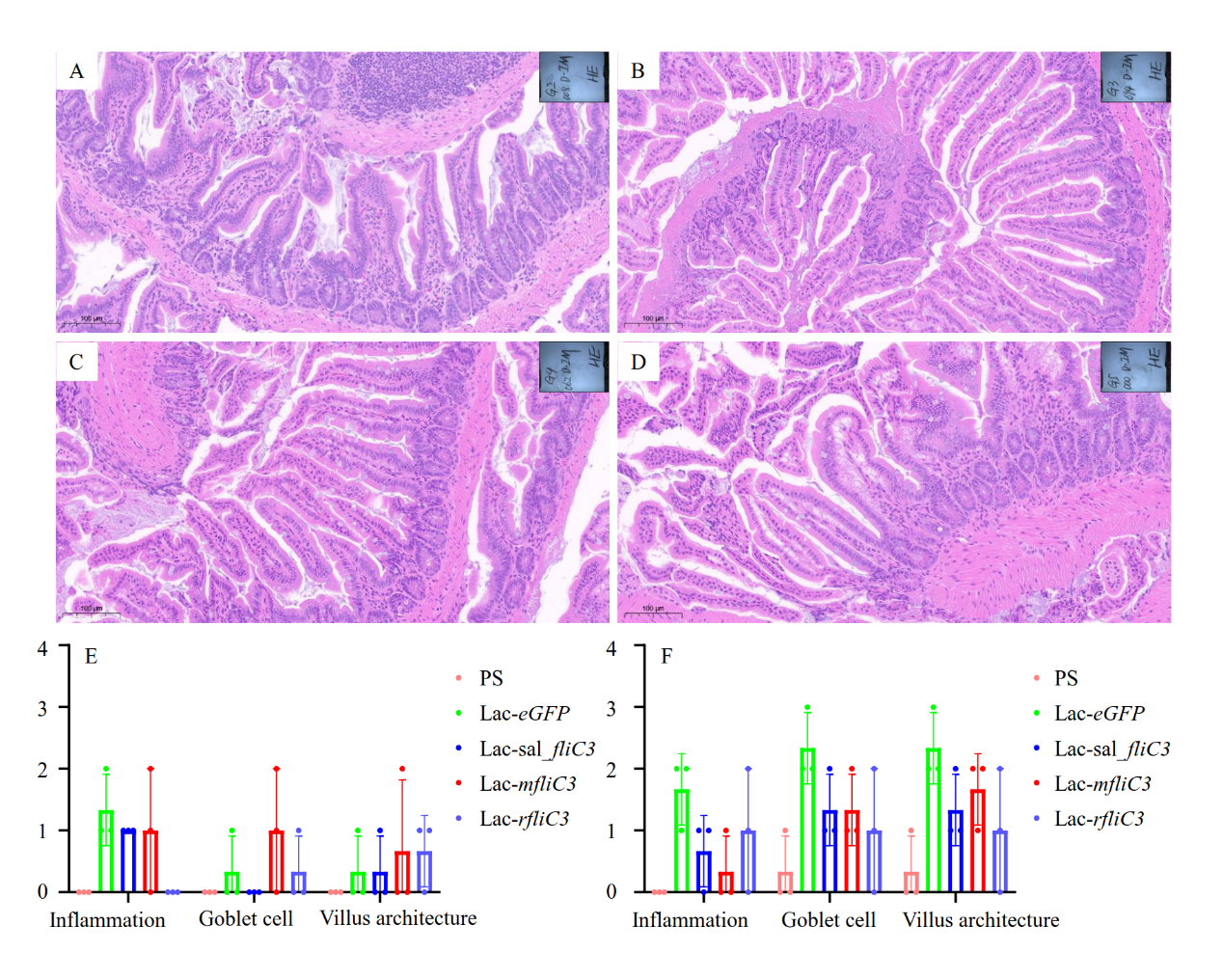
**

**Supplementary Figure 7. Subcellular localization of Lac-*mFliC3* in MODE-K murine intestinal epithelial cells.** MODE-K cells were incubated with Lac-*mFliC3* for 12 hours. Following PBS washing, triple immunofluorescence staining was performed using DAPI nuclear counterstain (blue), anti-mFliC3 antibody (green), and anti-LAMP2 endosomal marker (red). (A) DAPI staining results of cells; (B) Anti-SFB FliC3 antibody staining results of cells; (C) Anti-LAMP2 antibody staining results of cells; (D) Merged images of A, B, and C demonstrating co-localization signals (yellow) of FliC3 and LAMP2. Panel D shows merged fluorescence channels. Specimens were mounted in anti-fade medium and imaged by confocal microscopy (scale bar: 10 μm).


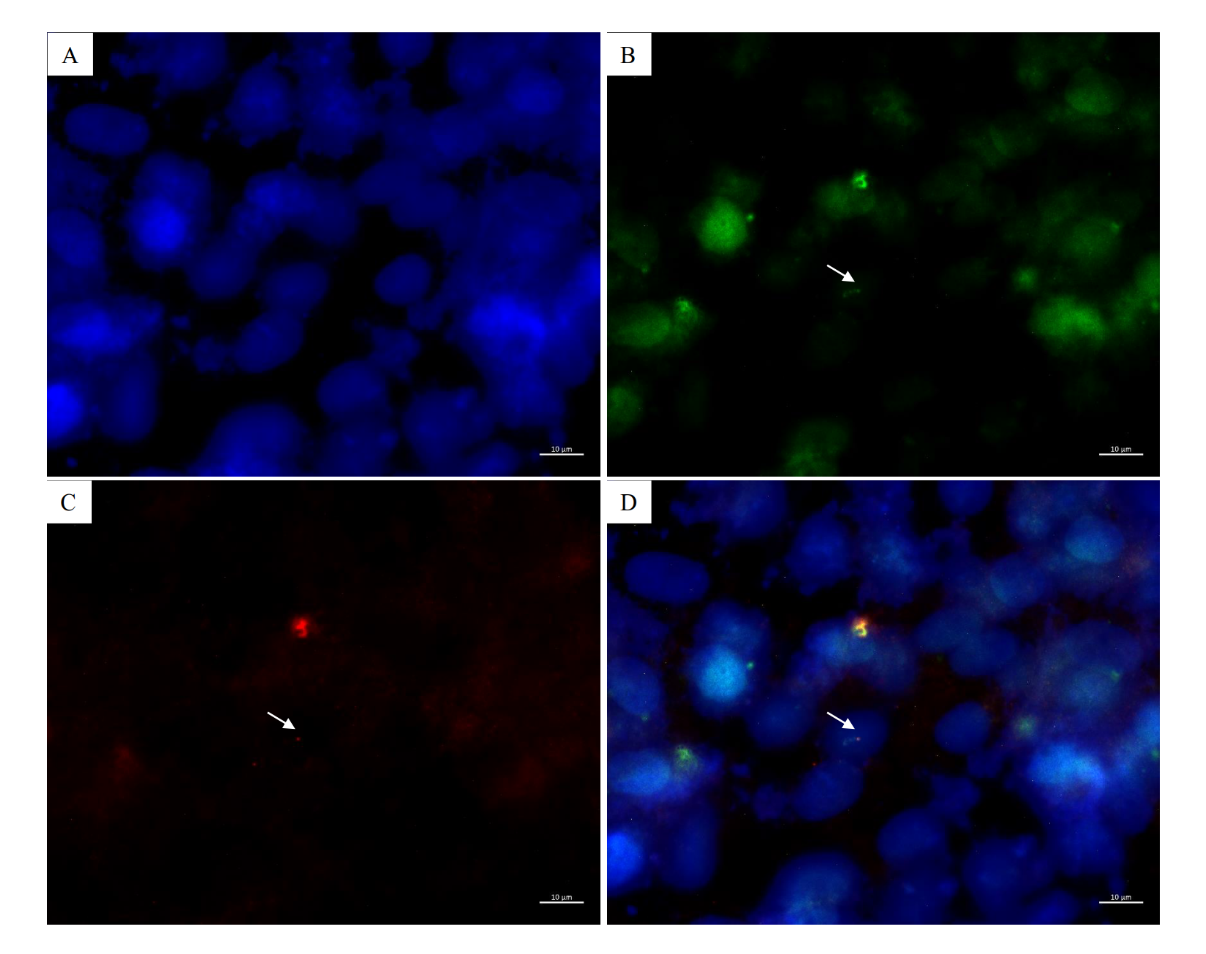


**Supplementary Figure 8. Growth curve of *E. coli* BW25113 and its derivative strains.** *E. coli* BW25113 and its derivative strains were inoculated into LB medium. Growth was assessed by measuring the optical density at 600 nm (OD₆₀₀) at the indicated time points.

**
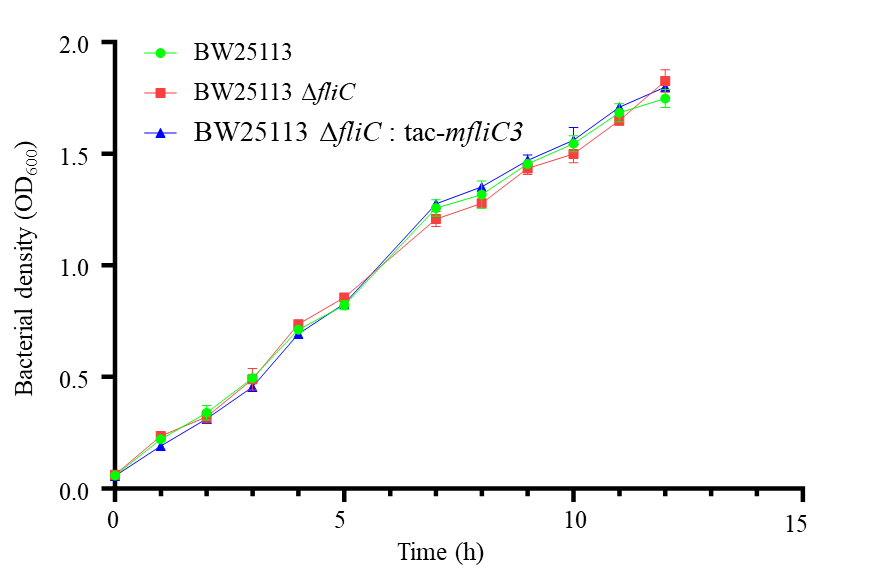
**

**Supplementary Figure 9. Change of *E. coli* morphology after *fliC3* editing.** Equal numbers of *E. coli* BW25113 and its derivative strains were spot-inoculated onto LB agar plates. Colony morphology was assessed after incubation for 24 h. WT, represents a wild-type *E. coli* strain BW25113; Δ *fliC*, represents an *E. coli* strain BW25113 that knocks out the *fliC* gene on the genome; Δ *fliC*:: *fliC3*, represent the *E. coil* strain BW25113 that the *fliC* gene on the genome was replaced with SFB *mfliC3*.


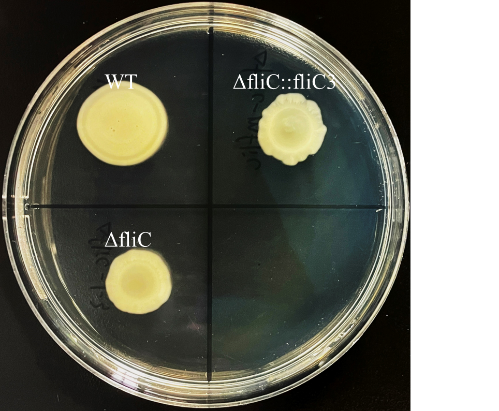


**Supplementary Figure 10. Verification the genome edited strain using primers *mflic*-JD-F and *mflic*-JD-R.** Genomic DNA was extracted from both *E. coli* BW25113 ΔfliC::tac-mFliC3 and the parental BW25113 strain for PCR analysis. M represents DNA ladder; Lane 1 represents the genome DNA of BW25113 *△fliC*:*tac-mfliC3* was used as PCR template，Lane 2 represents the genome DNA of wild type *E. coli* was used as PCR template.


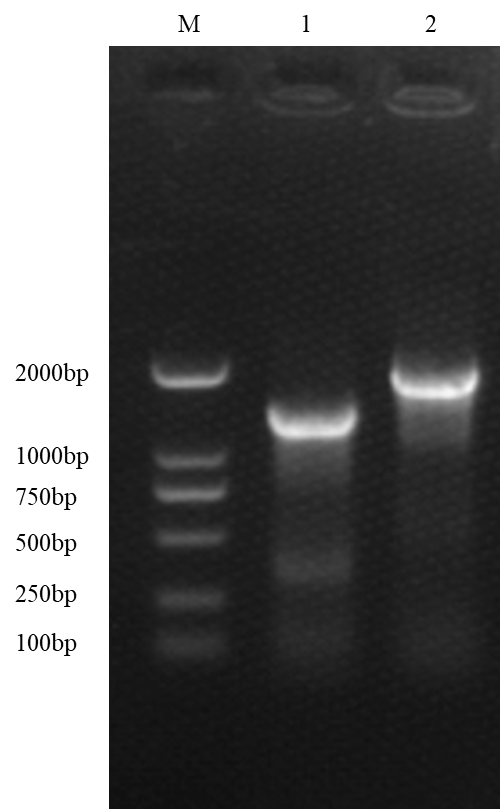


**Supplementary Figure 11. Detection of mFliC3 in pull-down assays.** Endophili A2 protein was purified from transfected 293T cells. Pull-down binding assays were performed using mFlC3 incubated with purified Endophili A2 protein (Lane 1), Magnetic Anti-FLAG Beads (Lane 2, negative control), or Endophilin A2-conjugated Magnetic Anti-FLAG Beads (Lane 3). mFliC3 was detected by immunoblotting using rabbit anti-mflC3 polyclonal antibody, HRP-conjugated goat anti-rabbit IgG, and visualized with a high-sensitivity ECL chemiluminescence kit. M: Protein molecular weight marker.

**
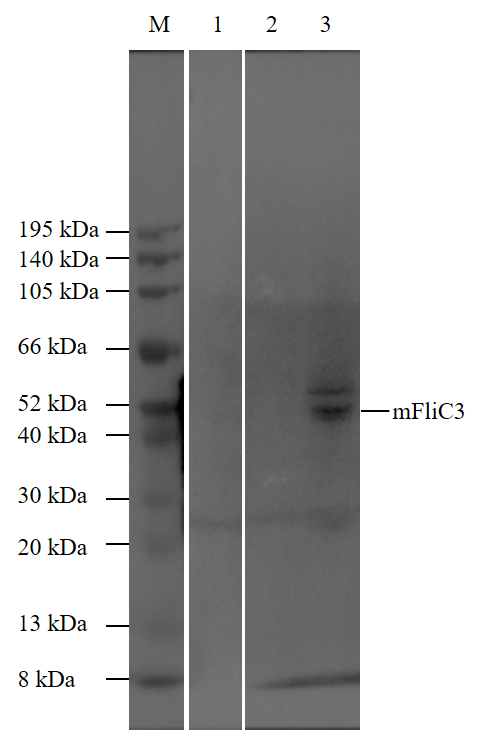
**

**Supplementary Table 1. List of primers for genome editing in *E. coli***

| Primer name | Primer Sequence |
| --- | --- |
| *flic*-up-F | 5-AATGCAGTATTGGCGGTCTGGAA-3 |
| *flic*-down-R | 5-TAGCAGATCGTCAAGTTCCACGC-3 |
| *flic*-up-R | 5-TAATCAGGTTACAACGAGATTCGTTATCCTATATTGCAAG-3 |
| *flic*-down-F | 5-ATAGGATAACGAATCTCGTTGTAACCTGATTAACTGAGAC-3 |
| *flic*-sgRNA | 5-GATACCGTCGTTGGCGTTAC-3 |
| *mflic*-down-F | 5-caccaccaccaccacTAATCGTTGTAACCTGATTAACTGAGAC-3 |
| *mflic*-up-R | 5-ccgatgattaattgtcaaGATTCGTTATCCTATATTGCAAG-3 |
| *mflic*-F | 5-ATATAGGATAACGAATCttgacaattaatcatcggctcgta-3 |
| *mflic*-R | 5-gtggtggtggtggtggtgTCTTAAGATTGAAAGAACTTG-3 |
| 28a-*tac*-F | 5-TATACCATGGAAGCTTACATGATAATTAATCACAATATG-3 |
| 28a-*tac*-R | 5-CGATGATTAATTGTCAAGCGCAACGCAATTAATGTAAGTT-3 |
| *Tac*-28a-F | 5-TTAATTGCGTTGCGCTTGACAATTAATCATCGGCTCGTAT-3 |
| *Tac*-28a-R | 5-GTGATTAATTATCATGTAAGCTTCCATGGTATATCTCCTT-3 |
| *mflic*-JD-F | 5-AGCGGGAATAAGGGGCAGAGAAA-3 |
| *mflic*-JD-R | 5-GGGAAGCACGTTGCTGACAAATT-3 |

**Supplementary Table 2. KEGG enrichment analysis of DEGs in the ileum of Lac-*sal_fliC3* group.**

| KEGG ID | Term description | No. DEGs | No. Background genes | FDR |
| --- | --- | --- | --- | --- |
| mmu05150 | Staphylococcus aureus infection | 10 | 108 | 0.000 |
| mmu05202 | Transcriptional misregulation in cancer | 9 | 191 | 0.000 |
| mmu04621 | NOD-like receptor signaling pathway | 7 | 193 | 0.000 |
| mmu04612 | Antigen processing and presentation | 5 | 75 | 0.000 |
| mmu04672 | Intestinal immune network for IgA production | 4 | 41 | 0.001 |
| mmu05145 | Toxoplasmosis | 5 | 106 | 0.001 |
| mmu05310 | Asthma | 3 | 24 | 0.003 |
| mmu04640 | Hematopoietic cell lineage | 4 | 90 | 0.008 |
| mmu05322 | Systemic lupus erythematosus | 4 | 93 | 0.008 |
| mmu00591 | Linoleic acid metabolism | 3 | 48 | 0.016 |
| mmu05330 | Allograft rejection | 3 | 51 | 0.017 |
| mmu05332 | Graft-versus-host disease | 3 | 50 | 0.017 |
| mmu04940 | Type I diabetes mellitus | 3 | 58 | 0.021 |
| mmu05321 | Inflammatory bowel disease | 3 | 60 | 0.022 |
| mmu05140 | Leishmaniasis | 3 | 66 | 0.026 |
| mmu05320 | Autoimmune thyroid disease | 3 | 67 | 0.026 |
| mmu04514 | Cell adhesion molecules | 4 | 157 | 0.028 |
| mmu04662 | B cell receptor signaling pathway | 3 | 74 | 0.030 |
| mmu05416 | Viral myocarditis | 3 | 74 | 0.030 |
| mmu05323 | Rheumatoid arthritis | 3 | 84 | 0.038 |
| mmu00590 | Arachidonic acid metabolism | 3 | 87 | 0.039 |
| mmu04658 | Th1 and Th2 cell differentiation | 3 | 86 | 0.039 |
| mmu00592 | alpha-Linolenic acid metabolism | 2 | 24 | 0.040 |

**Supplementary Table 3. KEGG enrichment analysis of DEGs in the hepatic of Lac-*rfliC3* group.**

| KEGG ID | Term description | No. DEGs | No. Background genes | FDR |
| --- | --- | --- | --- | --- |
| mmu05221 | Acute myeloid leukemia | 6 | 70 | 0.67 |
| mmu04933 | AGE-RAGE signaling pathway in diabetic complications | 10 | 99 | 0.74 |
| mmu05146 | Amoebiasis | 9 | 102 | 0.68 |
| mmu04210 | Apoptosis | 8 | 135 | 0.51 |
| mmu04024 | cAMP signaling pathway | 10 | 206 | 0.42 |
| mmu05210 | Colorectal cancer | 6 | 86 | 0.58 |
| mmu04625 | C-type lectin receptor signaling pathway | 7 | 109 | 0.54 |
| mmu04060 | Cytokine-cytokine receptor interaction | 19 | 280 | 0.57 |
| mmu05169 | Epstein-Barr virus infection | 16 | 214 | 0.61 |
| mmu05418 | Fluid shear stress and atherosclerosis | 13 | 142 | 0.7 |
| mmu04068 | FoxO signaling pathway | 9 | 129 | 0.58 |
| mmu04640 | Hematopoietic cell lineage | 9 | 90 | 0.74 |
| mmu05161 | Hepatitis B | 9 | 160 | 0.49 |
| mmu05160 | Hepatitis C | 11 | 158 | 0.58 |
| mmu04066 | HIF-1 signaling pathway | 7 | 110 | 0.54 |
| mmu05163 | Human cytomegalovirus infection | 13 | 237 | 0.48 |
| mmu04657 | IL-17 signaling pathway | 12 | 89 | 0.87 |
| mmu05321 | Inflammatory bowel disease | 6 | 60 | 0.74 |
| mmu05164 | Influenza A | 11 | 166 | 0.56 |
| mmu04931 | Insulin resistance | 10 | 109 | 0.7 |
| mmu04630 | JAK-STAT signaling pathway | 10 | 165 | 0.52 |
| mmu05167 | Kaposi sarcoma-associated herpesvirus infection | 10 | 210 | 0.41 |
| mmu05134 | Legionellosis | 9 | 60 | 0.91 |
| mmu05140 | Leishmaniasis | 6 | 66 | 0.69 |
| mmu04670 | Leukocyte transendothelial migration | 7 | 113 | 0.53 |
| mmu05144 | Malaria | 7 | 52 | 0.87 |
| mmu04010 | MAPK signaling pathway | 21 | 287 | 0.6 |
| mmu05162 | Measles | 12 | 140 | 0.67 |
| mmu05206 | MicroRNAs in cancer | 12 | 159 | 0.61 |
| mmu04064 | NF-kappa B signaling pathway | 13 | 99 | 0.85 |
| mmu04621 | NOD-like receptor signaling pathway | 14 | 193 | 0.6 |
| mmu04932 | Non-alcoholic fatty liver disease | 10 | 147 | 0.57 |
| mmu04380 | Osteoclast differentiation | 10 | 119 | 0.66 |
| mmu05212 | Pancreatic cancer | 7 | 72 | 0.72 |
| mmu04928 | Parathyroid hormone synthesis, secretion and action | 9 | 106 | 0.67 |
| mmu05200 | Pathways in cancer | 21 | 527 | 0.34 |
| mmu05133 | Pertussis | 7 | 73 | 0.72 |
| mmu04151 | PI3K-Akt signaling pathway | 17 | 353 | 0.42 |
| mmu04141 | Protein processing in endoplasmic reticulum | 10 | 169 | 0.51 |
| mmu05205 | Proteoglycans in cancer | 11 | 196 | 0.49 |
| mmu05323 | Rheumatoid arthritis | 7 | 84 | 0.66 |
| mmu04659 | Th17 cell differentiation | 8 | 101 | 0.63 |
| mmu04668 | TNF signaling pathway | 15 | 112 | 0.86 |
| mmu04620 | Toll-like receptor signaling pathway | 11 | 97 | 0.79 |
| mmu05145 | Toxoplasmosis | 7 | 106 | 0.56 |
| mmu05202 | Transcriptional misregulation in cancer | 13 | 191 | 0.57 |
| mmu05152 | Tuberculosis | 10 | 168 | 0.51 |
